# The Role of 5-Hydroxymethylcytosine as a Potential Epigenetic Biomarker in a Large Series of Thyroid Neoplasms

**DOI:** 10.1007/s12022-024-09800-9

**Published:** 2024-01-29

**Authors:** Sule Canberk, João Gonçalves, Elisabete Rios, Antónia A. Povoa, Ebru Tastekin, Manuel Sobrinho-Simões, Aysun Uguz, Ozlem Aydin, Umit Ince, Paula Soares, Valdemar Máximo

**Affiliations:** 1 Instituto de Investigação e Inovação em Saúde (i3S), University of Porto, Rua Alfredo Allen 208, 4200-135 Porto, Portugal; 2 Institute of Molecular Pathology and Immunology of the University of Porto (Ipatimup), Rua Júlio Amaral de Carvalho 45, 4200-135 Porto, Portugal; 3 Abel Salazar Institute of Biomedical Sciences (ICBAS), University of Porto, Rua de Jorge Viterbo Ferreira 228, 4050-313 Porto, Portugal; 4 Faculty of Medicine of the University of Porto (FMUP), Alameda Professor Hernâni Monteiro, 4200-319 Porto, Portugal; 5 Department of Pathology, Faculty of Medicine of the University of Porto, Alameda Prof. Hernâni Monteiro, 4200-319 Porto, Portugal; 6 Department of General Surgery, Centro Hospitalar de Vila Nova de Gaia/Espinho (CHVNG/E), 4434-502 Vila Nova de Gaia, Portugal; 7 Medical Faculty, Department of Pathology, Trakya University, Edirne, Turkey; 8 Medical Faculty, Department of Pathology, Çukurova University, Adana, Turkey; 9 Department of Pathology, School of Medicine, Acibadem Mehmet Ali Aydinlar University, Istanbul, Turkey

**Keywords:** Thyroid tumors, Hürthle cell tumor, Oncocytic cell tumors, Epigenetics, 5hmC

## Abstract

Cytosine modifications at the 5-carbon position play a critical role in gene expression regulation and have been implicated in cancer development. 5-Hydroxymethylcytosine (5hmC), arising from 5-methylcytosine (5-mC) oxidation, has shown promise as a potential malignancy marker due to its depletion in various human cancers. However, its significance in thyroid tumors remains underexplored, primarily due to limited data. In our study, we evaluated 5hmC expression levels by immunohistochemistry in a cohort of 318 thyroid tumors. Our analysis revealed significant correlations between 5hmC staining extension scores and nodule size, vascular invasion, and oncocytic morphology. Nuclear 5hmC staining intensity demonstrated associations with focality, capsule status, extrathyroidal extension, vascular invasion, and oncocytic morphology. Follicular/oncocytic adenomas exhibited higher 5hmC expression than uncertain malignant potential (UMP) or noninvasive follicular thyroid neoplasms with papillary-like nuclear features (NIFTP), as well as malignant neoplasms, including papillary thyroid carcinomas (PTCs), oncocytic carcinomas (OCAs), follicular thyroid carcinomas (FTCs), and invasive encapsulated follicular variants of PTC (IEFV-PTC). *TERT* promoter mutation cases showed notably lower values for the 5hmC expression, while *RAS* (*H*,* N*, or* K*) mutations, particularly *HRAS* mutations, were associated with higher 5hmC expression. Additionally, we identified, for the first time, a significant link between 5hmC expression and oncocytic morphology. However, despite the merits of these discoveries, we acknowledge that 5hmC currently cannot segregate minimally invasive from widely invasive tumors, although 5hmC levels were lower in wi-FPTCs. Further research is needed to explore the potential clinical implications of 5hmC in thyroid tumors.

## Introduction

Alterations in global 5hmC levels have emerged as an epigenetic marker of cancer. The loss of 5hmC has already been reported in some cancer models, such as melanoma, brain tumors, hematologic malignancies and bile duct, lung, ovarian, and hepatocellular carcinomas, as it acts as a promoter of neoplastic transformation and progression of neoplastic cells [1–7]. 

The role of 5hmC in thyroid tumors remains to be clarified. There are few studies on this role, but some data have begun to emerge, based on relatively small and unrepresentative series, regarding tumor pattern diversity [8–12]. In a cohort that included only papillary thyroid carcinomas (PTCs) (*n* = 88) and multinodular goiter (MNG) (*n* = 20) cases, Tong et al. described a decreased level of 5hmC in PTC tumor samples from patients with lymph node metastasis compared to PTC tumor samples from patients without lymph node metastasis [8]. Seok et al. showed a significant reduction in 5hmC expression in anaplastic thyroid carcinoma (ATC) samples in comparison with PTC and follicular thyroid carcinoma (FTC) samples in a cohort of 24 cases [9]. The same group later focused on follicular patterned thyroid tumors in a series with 40 cases and reported decreased 5hmC expression in the invasive/infiltrative follicular variant of PTC (iFVPTC) with < 1% papillary structure together with frequent regional lymph node involvement and *BRAF V600E* mutation when compared with other follicular patterned neoplasms without any papillary structures [10]. Oishi et al., through ELISA and IHC, analyzed the global 5hmC level in 85 thyroid carcinomas and suggested that 5hmC loss can be used as an epigenetic hallmark of *TERT* promoter-mutated PTCs and ATCs [11]. Last, Hysek et al. investigated the role of 5hmC as a potential predictor of *TERT* promoter mutation status in 29 cases of follicular patterned thyroid tumors. The study found that 5hmC immunohistochemistry has low sensitivity but high specificity, leading the authors to conclude that 5hmC is not a reliable marker for identifying *TERT* promoter mutations in this group of tumors [12].

To better understand the diagnostic and prognostic role of 5hmC in thyroid tumorigenesis, our group collected a multi-institutional comprehensive dataset. A total of 318 cases representing a wide variety of benign, low-risk, and malignant thyroid neoplasms were analyzed by immunohistochemistry for 5hmC expression. The 5hmC levels were compared among the different tumor groups, considering their clinical, demographic, and genomic characteristics, to validate patterns of 5hmC expression and its potential use in the clinical management of thyroid neoplasms.

## Materials and Methods

### Patient Tissue Samples

Three hundred eighteen patients were enrolled from six different tertiary centers: Centro Hospitalar de Vila Nova de Gaia e Espinho (*n* = 183), Hospital São João (*n* = 51), Trakya University (*n* = 27), IPATIMUP (*n* = 25), Çukurova University (*n* = 18), and Acibadem University (*n* = 14). The demographic and clinicopathological data of the patients were retrospectively collected from the histopathological reports and clinical databases. The histology of all tumor samples was reviewed independently by an endocrine pathologist (S.C.), and thyroid tumor classification was performed based on the 5th edition of the WHO Classification of Endocrine and Neuroendocrine Tumors [13]. From the 318 patients, 318 tumors were evaluated, and the following clinicopathological characteristics were collected: sex, age, tumor size, tumor location, capsule status, extrathyroidal extension, lymphatic invasion, vascular invasion, oncocytic morphology, and biological behavior. Tumors with oncocytic morphology were defined when more than 75% of the neoplastic cell population had oncocytic cytology.

Previous genetic characterization was obtained for a sample of patients (*n* = 183) [14]. This study protocol was approved by the Ethics Committee for Health (CES) of the Hospital Center of São João/Faculty of Medicine of the University of Porto (CES 66–19). The entire study was conducted in compliance with the Helsinki Declaration and national ethical norms (Law no. 12/2005) [14].

### Immunohistochemistry (IHC)

Four-micrometer-thick tissue sections from representative tumor blocks were deparaffinized and rehydrated in a graded series of ethanol solutions, as previously described [14]. Heat-induced antigen retrieval was performed using ethylenediaminetetraacetic acid buffer with a pH of 9.0 (LabVision Corporation, Fremont, CA, USA). Endogenous peroxidase activity and nonspecific binding were blocked with UltraVision Hydrogen Peroxide Block and UltraVision Block reagents (10 min), respectively (UltraVision Quanto Detection System HRP DAB, Thermo Scientific/Lab Vision, Fremont, USA) [18]. The sections were then incubated in a humidified chamber with a rabbit polyclonal antibody against 5-hydroxymethylcytosine (cat. no. 39791; Active motif, Carlsbad, US) at a 1:10,000 dilution, according to the manufacturer’s specifications. In each run, previously tested glioma samples and samples subjected to an immunostaining reaction lacking the primary antibody were used as positive and negative controls, respectively. Mayer’s hematoxylin was used to counterstain all sections. It is important to highlight that the choice of a 1:100,000 dilution of the antibody was preceded by several dilution tests that ranged from 1:2500 to 1:10,000. The tests were carried out on formalin-fixed paraffin-embedded (FFPE) samples derived from several tissues and tumors from different organs, employing both manual and automated (BenchMark ULTRA) IHC methods. The intensity and extension of 5-hmC staining did not vary significantly with dilution, but the staining contrast was much better at higher dilutions, especially in some types of tumors, notably thyroid and kidney. The quality and intensity of 5-hmC staining in the samples were independent of the age of the block.

### Evaluation of Immunohistochemical Staining

Immunoreactivity was present at the nucleus of cancer cells and was semi-quantitatively evaluated for each tumor sample. Immunostaining evaluation (by S.C. and V.M.) was based on the intensity and distribution of the staining, without knowledge of any clinical information of the cases. Two authors jointly evaluated the 5hmC immunoreactivity, utilizing a multi-headed microscope for synchronous examination of the cases. This collaborative approach of immediate, concurrent observation ensured a consistent and collective agreement in our findings. The 5hmC staining intensity was scored as absent (0), weak (1), moderate (2), or strong (3). Weak staining was not discernible on a low-power view (×4 objective), while moderate and strong staining was easily detectable on low-power magnification. The extent of nuclear staining was classified into four groups: < 25%, 26–50%, 51–75%, and 76–100%. The H staining score (H-score) was applied to evaluate the immune expression of 5hmC. To calculate the H-score (range 0–300), the following formula was used: H‐score = 0 × (% of cells staining with intensity value 0) + 1 × (% of cells staining with intensity value 1) + 2 × (% of cells staining with intensity value 2) + 3 × (% of cells staining with intensity value 3) [15]. For statistical purposes, the score was used either as a continuous variable or divided in a binary fashion: 0–150; 151–300.

### DNA Extraction

After H&E-guided microdissection, DNA from FFPE tissues was obtained from 10 µm sections. The GRS Genomic DNA Kit BroadRange was used for DNA extraction according to the manufacturer’s (GRiSP Research Solutions, Porto, Portugal) instructions, as previously described [14].

### PCR and Sanger Sequencing Analysis

Analysis of the mutations in hotspot regions of *BRAF* (codon 600), *RAS* [*NRAS* (codon 61), *HRAS* and *KRAS* (codons 12, 13, and 61)], and *TERTp* (− 124 and − 146 promoter regions) were previously performed in a subset of PTCs [14]. Briefly, 25–50 ng of genomic DNA was amplified for each genomic region under study utilizing the QIAGEN multiplex PCR kit (QIAGEN, Hilden, Germany). PCR products were purified and subsequently sequenced by Sanger using the ABI Prism Big Dye Terminator kit v3.1 Cycle Sequencing (Fisher Scientific Applied Biosystems^®^, Portsmouth, NH, USA). Whenever mutations were identified, a new and independent analysis was conducted to validate the presence of the mutation [14].

### Statistical Analysis

IBM SPSS Statistics V.26 (IBM, New York, New York, USA) was used for the statistical analysis. The data were reported using the following metrics: absolute frequency, percentage, mean Std, and median IR 2. Chi-square, Fisher’s exact, and Pearson’s correlation tests were used for univariate analysis and correlation analysis. Independent-sample *t* tests, Mann–Whitney *U* tests, one-way ANOVA, and Tukey’s HSD tests were applied whenever possible. A two-tailed *p* value of less than 0.05 was considered statistically significant.

## Results

### Cohort Characteristics

**Fig. 1:**
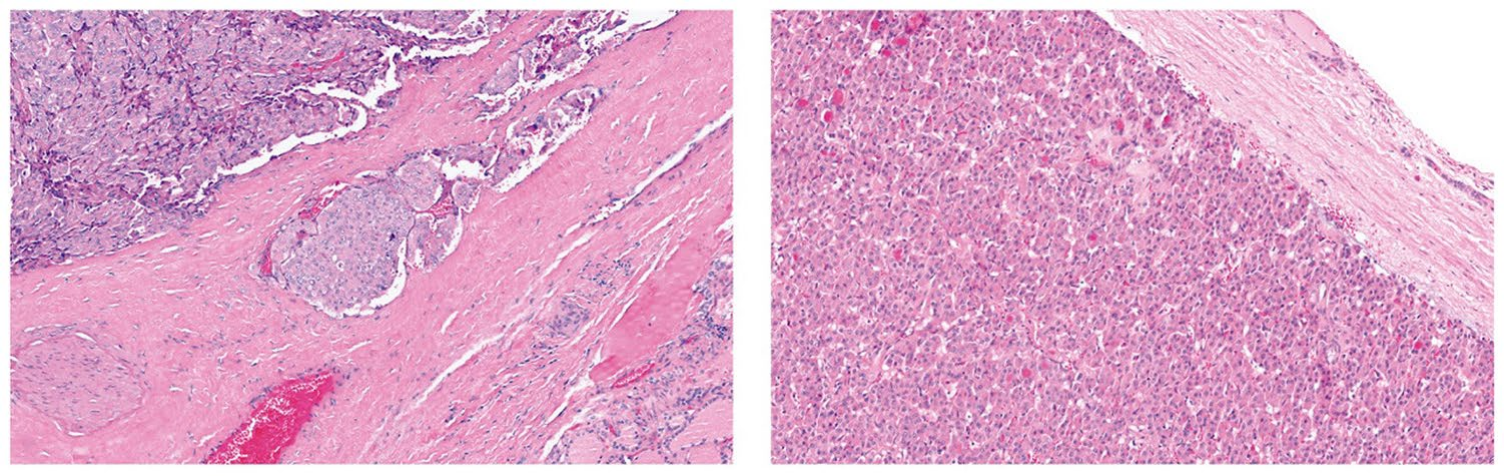
Microscope images of H&E stained tumors are displayed side by side. The image on the left shows an encapsulated angioinvasive oncocytic carcinoma (eai-OCA), and the image on the right features oncocytic adenoma (OA)

The cohort characteristics are summarized in Table 1. There were 253 (79.6%) females and 65 (20.4%) males. The ages ranged between 16 and 99 years, with a mean age of 51.6 (Table 1). After histological revision, the 318 thyroid tumors were classified as follows: 14 oncocytic adenomas (OAs), 15 follicular adenomas (FAs), 3 well-differentiated tumors with uncertain malignant potential (WDT-UMPs), 5 follicular tumors with uncertain malignant potential (FT-UMPs), 18 noninvasive follicular thyroid neoplasms with papillary-like nuclear features (NIFTP), 14 invasive encapsulated follicular variants of PTC (IEFV-PTC), 198 PTCs [114 classic subtypes of PTC (C-PTC), 33 infiltrative follicular variants of PTC (iFV-PTC), 27 hobnail subtypes of PTC (HN-PTC), 10 oncocytic subtypes of PTC (O-PTC), 4 Warthin-like PTCs (WL-PTC), 4 tall cell subtypes of PTC (TC-PTC), 3 solid/trabecular subtypes of PTC (ST-PTC), 2 encapsulated PTCs (E-PTC), and 1 diffuse sclerosing subtype of PTC (DS-PTC)], 12 follicular thyroid carcinomas (FTCs), and 39 oncocytic carcinomas (OCAs). The median nodule size was 2.0 ± 0.8 cm. Regarding focality, 193 (64.8%) of the tumors were unifocal, 105 (35.2%) were multifocal, 233 (81.1%) were unilateral, and 54 (18.8%) were bilateral. The capsule status was invasive/infiltrative in 189 (73.5%) cases (Table 1). Extrathyroidal extension was evidenced in 101 (31.8%) cases, of which only four had gross extension. Lymphatic invasion was present in 56 (17.7%) samples, while vascular invasion was present in 41 (12.9%). Oncocytic morphology was present in 105 (33.1%) tumors: 14 OAs, 39 OCAs, 42 subtypes of PTC (26 hobnail, 10 oncocytic, 4 Warthin-like, and 2 tall cells), eight UMPs, and two NIFTPs (Fig. 1). In total, the series included 251 (78.9%) malignant, 29 (9.1%) benign, and 38 (11.9%) low-risk neoplasms. Distant metastases were demonstrated in 4 (2.8%) cases (Table 1).

**Table 1:** Cohort description: patient characteristics, clinicopathological features, genetical evaluation, and scoring systems

**Characteristics**	***n***	**(%)**	***n* (total)**	**Characteristics**	***n***	**(%)**	***n* (total)**
**Gender**				**Distant metastasis**			145
Female	253	(79.6%)		Yes	4	(2.8%)	
Male	65	(20.4%)		No	141	(97.2%)	
**Age**				***TERT* mutation**			167
Mean ± std (years)	51.6 ± 16.5			Yes	12	(7.2%)	
**Size**				No	155	(92.8%)	
Median ± IR ÷ 2 (cm)	2.0 ± 0.8			***BRAF* mutation**			178
**Focality**			298	Yes	111	(62.3%)	
Unifocal	193	(64.8%)		No	67	(37.6%)	
Multifocal	105	(35.2%)		***NRAS* mutation**			178
**Laterality**			287	Yes	8	(4.5%)	
Unilateral	233	(81.1%)		No	170	(95.5%)	
Bilateral	54	(18.8%)		***HRAS* mutation**			97
**Capsule status**			257	Yes	9	(9.3%)	
Invasive/infiltrative	189	(73.5%)		No	88	(90.7%)	
No	68	(26.5%)		***KRAS* mutation**			93
**Extrathyroidal extension**			318	Yes	4	(4.3%)	
Minimal/major	101	(31.8%)		No	89	(95.7%)	
No	217	(68.2%)		**5hmC intensity**			318
**Lymphatic invasion**			316	0	37	(11.6%)	
Yes	56	(17.7%)		1	101	(31.8%)	
No	260	(82.3%)		2	80	(25.2%)	
**Vascular invasion**			317	3	100	(31.4%)	
Yes	41	(12.9%)		**5hmC extension**			318
No	276	(87.1%)		≤ 25	12	(3.8%)	
**Oncocytic morphology**			315	26–50	23	(7.2%)	
Yes	105	(33.1%)		51–75	29	(9.1%)	
No	212	(66.9%)		76–100	254	(79.9%)	
**Biological behavior**			318	**5hmC H-score**			318
Benign lesions	29	(9.1%)		≤ 150	132	41.5%	
Low-risk neoplasms	38	(11.9%)		151–300	186	58.5%	
Malignant neoplasms	251	(78.9%)

In a subgroup composed of 183 PTCs, mutational analysis was available [14], and mutations were found in 144 cases. These mutations included *BRAF* mutations in 111 (62.3%) cases, *TERT* mutations in 12 (7.2%) cases, *HRAS* mutations in 9 (9.3%) cases, *NRAS* mutations in 8 (4.5%) cases, and *KRAS* mutations in 4 (4.3%) cases.

### Association Between Pathological Characteristics of the Tumors and Intensity, Extension, and H-Score of 5hmC

A 5hmC staining extension score between 0 and 25% was found in 12 (3.8%) cases, a score between 25 and 50% was found in 23 (7.2%) cases, a score between 50 and 75% was found for 29 (9.1%) cases, and a score between 75 and 100% was found in 254 (79.9%) cases. Statistical significance was found in nodule size, vascular invasion, and oncocytic morphology in relation to 5hmC staining extension (Table 2).

**Table 2:** Analysis of immunohistochemical scoring of 5hmC in clinicopathological characteristics

**Pathological characteristics**	**Extension score 0 (0–25%)**	**Extension (score 1) (25–50%)**	**Extension (score 2) (50–75%)**	**Extension (score 3) (75–100%)**	***p*** **value**
**Size (cm)**	***n* (%)**	***n* (%)**	***n* (%)**	***n* (%)**	
< 4	11 (3.6)	18 (5.8)	18 (5.8)	219 (71.1)	0.003*
≥ 4	1 (0.3)	5 (1.6)	10 (3.2)	26 (8.4)	
**Vascular invasion**	***n* (%)**	***n* (%)**	***n* (%)**	***n* (%)**	
Yes	4 (1.3)	9 (2.8)	8 (2.5)	20 (6.3)	< 0.001*
No	8 (2.5)	14 (4.4)	21 (6.6)	233 (73.5)	
**Oncocytic morphology**	***n* (%)**	***n* (%)**	***n* (%)**	***n* (%)**	
Yes	5 (1.6)	15 (4.7)	15 (4.7)	70 (22.1)	** < 0.001**
No	7 (2.2)	7 (2.2)	14 (4.4)	184 (58.0)	

**Pathological characteristics (*n*)**	**Intensity score 0**	**Intensity score 1**	**Intensity score 2**	**Intensity score 3**	***p* value**
**Focality**	***n* (%)**	***n* (%)**	***n* (%)**	***n* (%)**	
Unifocal	19 (6.4)	54 (18.1)	54 (18.1)	66 (22.1)	0.053
Multifocal	13 (4.4)	44 (14.8)	21 (7.0)	27 (9.1)	
**Capsule status**	***n* (%)**	***n* (%)**	***n* (%)**	***n* (%)**	
Invasive/infiltrative	26 (10.1)	65 (25.3)	52 (20.2)	46 (17.9)	** < 0.001**
No	4 (1.6)	11 (4.3)	7 (2.7)	46 (17.9)	
**ETE**	***n* (%)**	***n* (%)**	***n* (%)**	***n* (%)**	
Minimal/major	18 (5.7)	43 (13.5)	32 (10.1)	8 (2.5)	** < 0.001**
No	19 (6.0)	58 (18.2)	48 (15.1)	92 (28.9)	
**Vascular invasion**	***n* (%)**	***n* (%)**	***n* (%)**	***n* (%)**	
Yes	14 (4.4)	11 (3.5)	5 (1.6)	11 (3.5)	** < 0.001**
No	23 (7.2)	90 (28.4)	74 (23.3)	89 (28.1)	
**Oncocytic morphology**	***n* (%)**	***n* (%)**	***n* (%)**	***n* (%)**	
Yes	22 (6.9)	29 (9.1)	17 (5.4)	37 (11.7)	** < 0.001**
No	14 (4.4)	72 (22.7)	63 (19.9)	63 (19.9)	
**Biological behavior**	***n* (%)**	***n* (%)**	***n* (%)**	***n* (%)**	
Benign lesions	0 (0.0)	3 (0.9)	0 (0.0)	26 (8.2)	** < 0.001**
Low-risk neoplasms	4 (1.2)	8 (2.5)	10 (3.1)	16 (5.0)	
Malignant neoplasms	33 (10.4)	90 (28.3)	70 (22.0)	58 (18.2)	

*p* values were determined using a chi-square test. Fisher’s exact test was used when indicated (*)

*p* values in bold indicate statistical significance

Nuclear 5hmC staining intensity was distributed as follows: 37 (11.6%) cases were negative, 101 (31.8%) cases were evaluated with intensity value 1 (weak staining), 80 (25.2%) cases were evaluated with intensity value 2 (moderate staining), and 100 (31.4%) cases were evaluated with intensity value 3 (strong staining). Statistical significance was observed for focality, capsule status, extrathyroidal extension, vascular invasion, oncocytic morphology, and biological behavior in relation to 5hmC intensity score values (Table 2).

The 5hmC H-score presented 132 (41.5%) samples belonging to the interval 0–150 and 186 (58.5%) to the 151–300 range. A significant association was identified between the 5hmC binary H-score evaluation and focality, laterality, capsule status, extrathyroidal extension, lymphatic invasion, vascular invasion, oncocytic morphology, and biological behavior (Table 3).

**Table 3:** Analysis of H-score evaluation of 5hmC in clinicopathological characteristics

**Pathological characteristics**	**H-score** **0–150**	**H-score** **151–300**	***p*** **value**
**Focality**	***n* (%)**	***n* (%)**	
**Unifocal**	68 (22.8)	125 (41.9)	**0.007**
**Multifocal**	54 (18.1)	51 (17.1)	
**Laterality**	***n* (%)**	***n* (%)**	
**Unilateral**	86 (30.0)	147 (51.2)	**0.043**
**Bilateral**	28 (9.8)	26 (9.0)	
**Capsule status**	***n* (%)**	***n* (%)**	
**Invasive/infiltrative**	86 (33.4)	103 (40.1)	** < 0.001**
**No**	14 (5.4)	54 (21.0)	
**ETE**	***n* (%)**	***n* (%)**	
**Minimal/major**	56 (17.6)	45 (14.2)	**0.001**
**No**	76 (23.9)	141 (44.3)	
**Lymphatic invasion**	***n* (%)**	***n* (%)**	
**Yes**	30 (9.5)	26 (8.2)	**0.004**
**No**	102 (32.2)	158 (49.8)	
**Vascular invasion**	***n* (%)**	***n* (%)**	
**Yes**	26 (8.2)	15 (4.7)	**0.002**
**No**	106 (33.4)	180 (56.8)	
**Oncocytic morphology**	***n* (%)**	***n* (%)**	
**Yes**	52 (16.4)	53 (16.7)	**0.037**
**No**	79 (24.9)	133 (42.0)	
**Biological behavior**	***n* (%)**	***n* (%)**	
**Benign lesions**	4 (1.2)	25 (7.9)	** < 0.001**
**Low-risk neoplasms**	10 (3.1)	28 (8.8)	
**Malignant neoplasms**	118 (37.1)	133 (41.8)	

*p* values were determined using a chi-square test

*p* values in bold indicate statistical significance

**Fig. 2:**
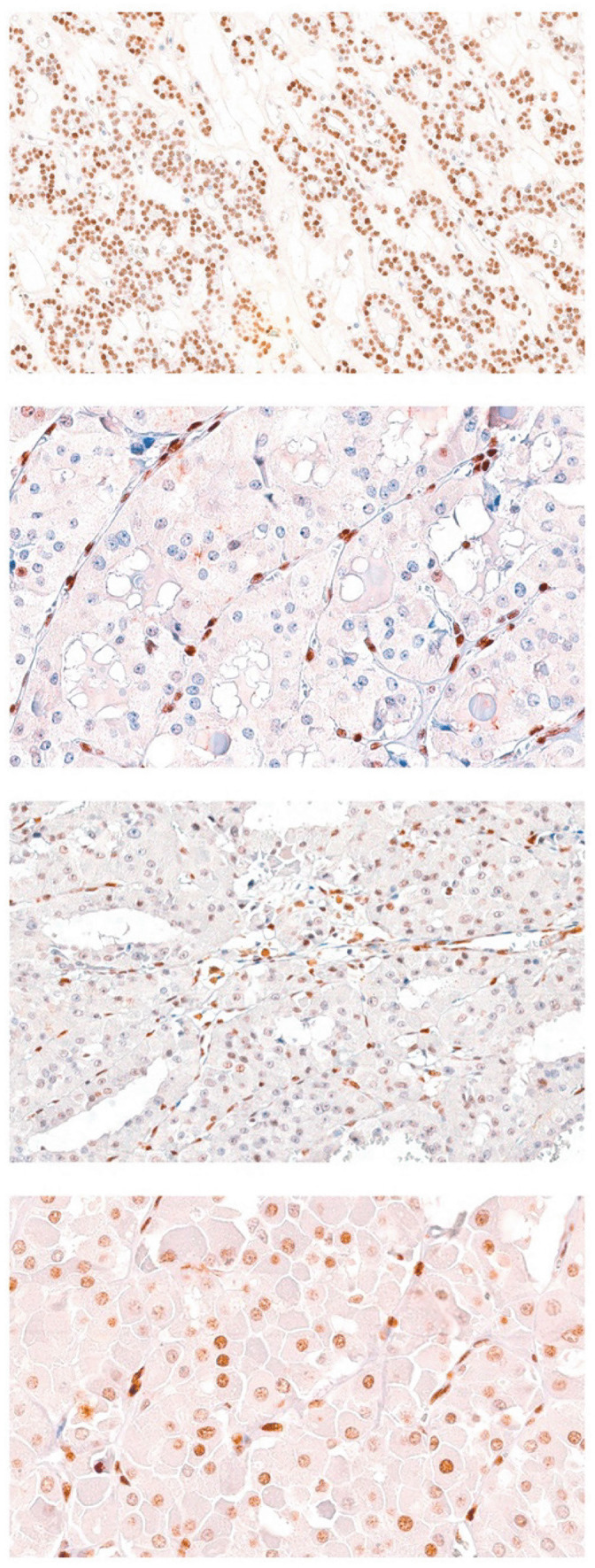
The images of 5hmC staining are sequentially arranged from top to bottom, each illustrating distinct staining patterns. The topmost image showcases prominent nuclear staining in follicular adenoma (FA), evidenced by an H-score of 270. This is followed by an image displaying markedly reduced nuclear staining in oncocytic carcinoma (OCA) with an H-score of 12. The third image from the top reveals relatively reduced and heterogeneous staining in another case of OCA with an H-score of 170. The bottom most image concludes the series with marked nuclear staining in oncocytic adenoma (OA) with an H-score of 230. In all cases, the varied staining patterns can be internally confirmed by the presence of positive 5hmC nuclear expression observed in the stromal cells of the tumor

A lower 5hmC H-score (evaluated as a continuous variable) was found in cases that presented the following clinicopathological characteristics: multifocality, bilaterality, invasive and infiltrative status of the capsule, minimal and major extrathyroidal extension, lymphatic invasion, vascular invasion, and oncocytic morphology (Table 4) (Fig. 2).

**Table 4:** Comparison of means of H-score evaluation of 5hmC for clinicopathological characteristics

**Pathological characteristics**	**Mean rank**	**Mann–Whitney test** ***p*** **value**
**Focality**		
**Unifocal**	158.06	**0.02**
**Multifocal**	133.77	
**Laterality**		
**Unilateral**	148.45	0.059
**Bilateral**	124.81	
**Capsule status**		
Invasive/infiltrative	114.11	** < 0.001**
No	170.38	
**ETE**		
**Minimal/major**	119.25	** < 0.001**
**No**	178.24	
**Lymphatic invasion**		
**Yes**	134.03	**0.027**
**No**	163.77	
**Vascular invasion**		
** Yes**	119.77	**0.003**
**No**	164.83	
**Oncocytic morphology**		
**Yes**	145.38	0.062
**No**	165.75	

**Pathological characteristics**	**Mean difference** (1st–2nd)	**Tukey HSD test** ***p* value**
**Biological behavior**		
**Benign–low-risk**	84.256	** < 0.001**
**Benign–malignant**	105.617	** < 0.001**
**Low-risk–malignant**	21.361	0.262

*p* values in bold indicate statistical significance

**Fig. 3:**
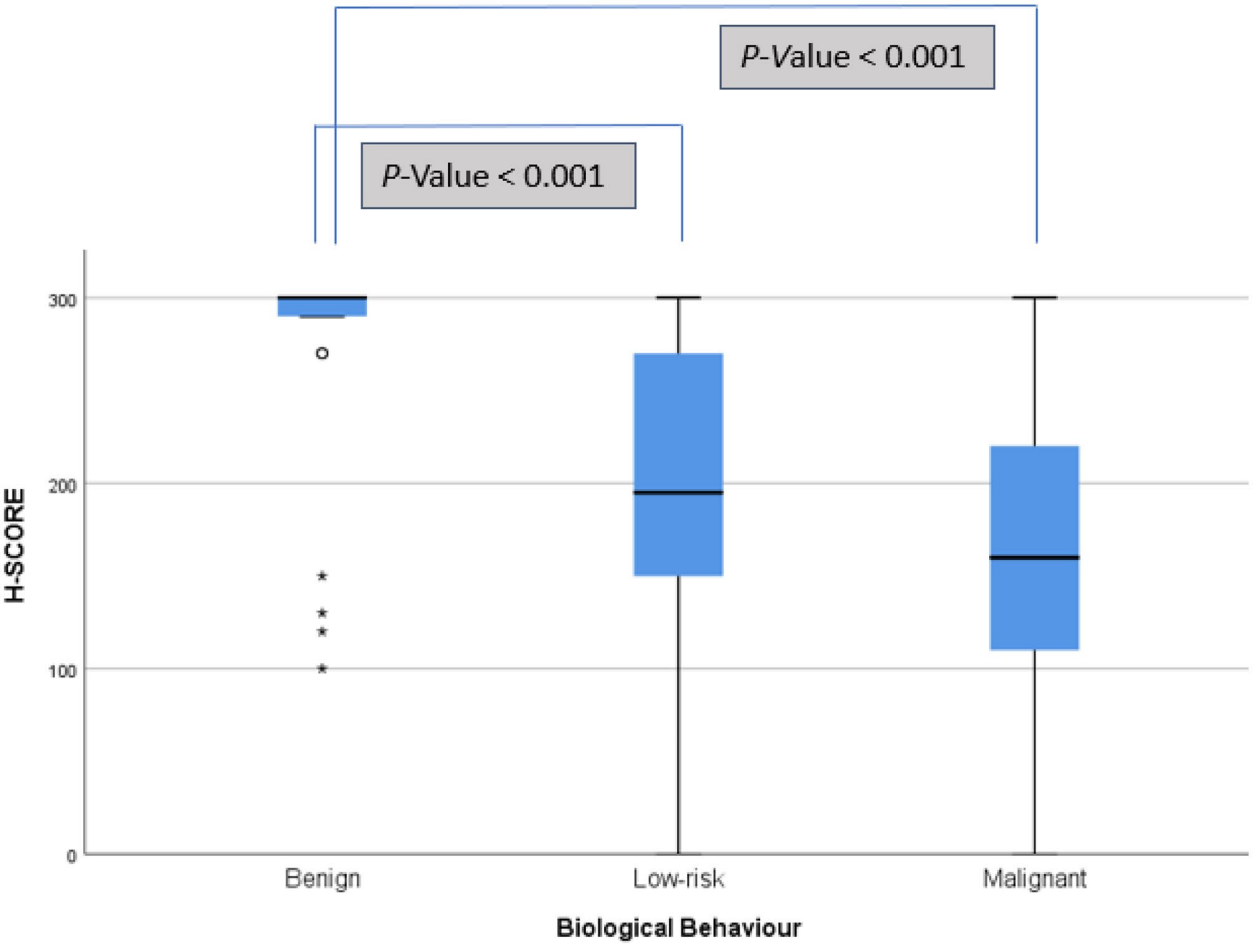
Box plot of H-score evaluation of 5hmC for biological behavior. Benign-FA/OA; low-risk-UMP (FT-UMP and WDT-UMP)/NIFTP; and malignant neoplasms-PTC, OCA, FTC, and IEFV-PTC

Regarding histological diagnosis and biological behavior, Fig. 3 shows that FA/OA showed a significantly higher H-score when compared with UMP (FT-UMP and WDT-UMP)/NIFTP and malignant neoplasms (PTC, OCA, FTC, and IEFV-PTC).

**Fig. 4:**
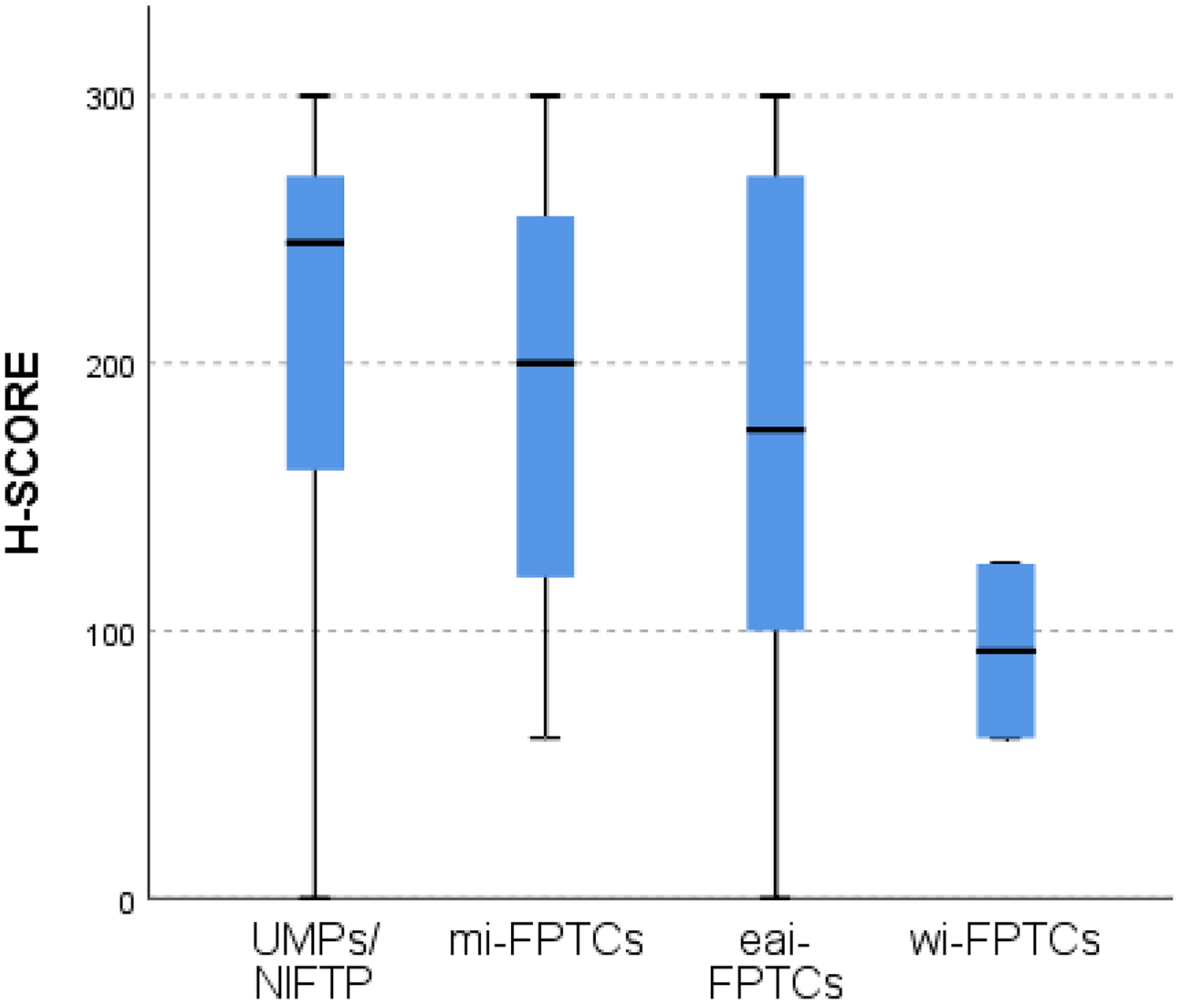
Box plot of H-score evaluation of 5hmC for low-risk neoplasms (UMP and NIFTP) and minimally invasive, encapsulated angioinvasive and widely invasive follicular pattern thyroid carcinomas (IEFV-PTC, FTC, and OCA)

There was no difference in 5hmC scores between low-risk neoplasms (UMP and NIFTP) and minimally invasive, encapsulated angioinvasive and widely invasive follicular pattern thyroid carcinomas, although 5hmC levels gradually decreased from UMP to wi-FPTCs (Fig. 4).

### Association Between the 5hmC H-Score and Tumor Pathological Characteristics and Tumor Molecular Status

A subgroup of 183 cases was also analyzed due to the availability of additional clinicopathological characteristics and molecular alterations. Of these 183 cases, 84 (45.9%) had H-score values between 0 and 150, and 99 (54.1%) cases had H-score values between 151 and 300. Cases presenting *TERT* promoter mutations showed significantly lower values for the 5hmC H-score (Table 5), whereas cases with *RAS* (*H*,* N*, or* K*) mutations (in general), specifically *HRAS* mutations, showed significantly higher values for the 5hmC H-score (Table 5). No significant correlation was found between *BRAF* mutation and 5hmC expression levels.

**Table 5:** Comparison of H-score evaluation of 5hmC with clinicopathological characteristics for a subgroup of 183 patients

Pathological characteristics	**H-score** **0–150**	**H-score** **151–300**	***p* value**
**Focality**	***n* (%)**	***n* (%)**	
**Unifocal**	42 (23.0)	72 (39.3)	**0.002**
**Multifocal**	42 (23.0)	27 (14.8)	
**Laterality**	***n* (%)**	***n* (%)**	
**Unilateral**	57 (33.1)	19 (11.0)	**0.003**
**Bilateral**	88 (51.2)	8 (4.7)	
**Psammoma bodies**	***n* (%)**	***n* (%)**	
**Present**	34 (18.8)	26 (14.4)	0.051
**Absent**	50 (27.6)	71 (39.2)	
**Distant metastasis**	***n* (%)**	***n* (%)**	
**Yes**	4 (2.8)	0 (0.0)	0.046*
**No**	64 (44.1)	77 (53.1)	
***TERT*** **mutation**	***n* (%)**	***n* (%)**	
**Yes**	9 (5.4)	3 (1.8)	**0.030**
**No**	66 (39.5)	89 (53.3)	
***HRAS*** **mutation**	***n* (%)**	***n* (%)**	
**Yes**	0 (0.0)	12 (12.4)	0.001*
**No**	39 (10.2)	46 (47.4)	
***RAS*** **mutation**	***n* (%)**	***n* (%)**	
**Yes**	7 (3.9)	18 (0.1)	0.058
**No**	75 (41.7)	80 (44.4)	

*p* values were determined using a chi-square test. Fisher’s exact test was used when indicated (*)

*p* values in bold indicate statistical significance

We assessed the impact of genetic mutations on the 5-hmC score using one-way ANOVA, categorizing mutation status as none, TERTp, BRAF, combined TERTp and BRAF, RAS, combined TERTp and RAS, and combined BRAF and RAS. No significant differences were found in 5-hmC H-scores (*p* = 0.337). Given the small sample size in some categories, caution is advised in interpreting the results. Despite the potential for confounders in multifactorial studies, the lack of significant variance in our results suggests that TERTp mutations do not significantly skew the data, reducing the likelihood of them being confounders.

## Discussion

Currently, the disruption of epigenetic landscapes is a well-recognized aspect of cancer, including abnormal DNA methylation patterns. DNA methylation and demethylation at the C-5 position occur in a cyclic manner involving many enzymes and proteins, influencing the regulation of gene transcription and expression. DNA methylation can be simply described as the transfer of a methyl group, to the 5′ position of a pyrimidine ring of cytosines in a CpG site. DNA demethylation can occur either passively or actively. Passive DNA demethylation can be described as the “dilution” of methylation levels after rounds of cellular replication due to the absence of methylation of the new DNA strand. Active DNA demethylation occurs via the activity of a group of enzymes called TET (ten-eleven translocation) family (TET1, TET2, and TET3) which realize active DNA demethylation through an iterative stepwise oxidization of 5-mC to 5hmC [15, 16]. Due to their roles in numerous metabolic pathways, 5-mC and 5hmC have both been investigated as potential epigenetic markers. However, unlike 5-mC, the content of 5hmC has revealed better stability and stronger robustness in different tissues. There is a growing body of literature showing the differences in 5hmC expression between cancerous and healthy tissues [17, 18]. A decreased nuclear level of 5hmC in comparison with normal tissue has been reported in some human cancers, such as melanoma, glioma, parathyroid carcinoma, hepatocellular carcinoma, cervical carcinoma, and hematologic malignancies [1, 4, 6, 19, 20]. Aligned with previous studies in other organs, our results in the present series of 318 thyroid tumors show that decreased or loss of expression of 5hmC was associated with malignant tumors when compared with benign tumors. However, the scores of 5hmC expression do not allow us to distinguish low-risk neoplasms from malignant neoplasms, confirming the borderline nature of these lesions.

In our study, information about tumor size was available for 308 cases and was grouped based on the cutoff value of 4 cm as suggested by the AJCC/TNM Staging System 8th edition [21]. We found a significant association between the extension score of 5hmC and tumor size (*p* value 0.003). Lymphatic invasion presented statistical significance in the evaluation of H-score, whereas vascular invasion represented statistical significance for 5hmC intensity, extension, and H-score. In accordance with these results, Tong et al. [8] compared the expression of 5-mC and 5hmC in 88 PTC, 20 MNG, and adjacent normal tissues based on a 3-tiered H-score evaluation. They found that PTC presented significantly less 5hmC than MNG. Curiously, for 5-mC, no difference was noted. These results support the sensitivity of 5hmC as a distinct epigenetic marker for establishing more stable DNA interactions over 5-mC, as shown in previous studies [15]. Tong et al. [8] also compared the H-score of PTC with and without lymph node metastasis and, in line with our results, found that the group with lymph node metastasis presented a lower score. In our series, loss of 5hmC expression was significantly associated with adverse pathological characteristics, such as minimal/major ETE, invasive/infiltrative capsule status, lymphatic invasion, vascular invasions, bilaterality, multifocality, and biological behavior (Table 6).

**Table 6:** 5hmC in thyroid neoplasm—a collated summary from discussed studies

**Study**	**No. of 5hmC-stained cases (*n*)**	**IHC evaluation method**	**5hmC antibody clone; manufacturer; dilution**	**Tumor histotype associations with 5hmC expression level**	**Clinicopathological characteristics’ associations with 5hmC expression levels**	**5hmC expression level in relation with gene mutations**
Tong et al. [8]	108	H-score	ab214728; monoclonal; Abcam; 1:200	Significantly lower in PTC vs. NG	Lymph node metastasis	No mutational analysis
	• 20 NG					
	• 88 PTC					
Seok et al. [10]	40	H-score	RM236; monoclonal; RevMAb Bioscience; 1:10,000	Preserved in groups I–III	Lymph node metastasis	No correlation analysis was performed between 5hmC expression levels and mutational status
	Only follicular-patterned thyroid neoplasm			Significantly lower in group IV		
	• **Group I:** 5 NIFTP					
	• **Group II:** 14 EFVPTC with capsular invasion					
	• **Group III:** 10 infiltrative FVPTC					
	• **Group IV:** 11 PTC with predominantly follicular pattern and focal papillae (< 1%)					
Oishi et al. [11]	Discovery group:	Only extension evaluation	Cat. no. 39791; polyclonal; Active Motif, 1:3000	No loss in PTC-wild-type *TERT*	NA	In PTC and ATC, *TERT* mutated cases showed lower 5hmC expression levels
	17	Nuclear staining in an estimated percentage of tumor cells		Global loss in PTC-*TERT* mutation		
	• 5 NTT			Global loss in ATC		
	• 5 PTC-wild-type *TERT*					
	• 4 PTC-*TERT* mutation					
	• 3 ATC					
	Validation group:					
	85					
	• 8 NTT					
	• 63 PTC (wild-type *TERT*)					
	• 10 PTCs (*TERT* mutation)					
Hysek et al. [12]	29	Only extension evaluation	RM236; monoclonal; RevMAb Bioscience; 1:1000	0 (0%) negative cases in 19 wild-type *TERT* mutated tumors	NA	No association of *TERT* promoter mutations with 5hmC expression levels
	Only follicular-patterned thyroid neoplasm	Nuclear staining in an estimated percentage of tumor cells				
	• 26 FTC			1 (10%) negative case in 10 *TERT* mutated tumors		
	• 2 FT-UMP					
	• 1 OCA		4D9; polyclonal; Epigentek; 1:2000	2 (11%) negative cases in 19 wild-type *TERT* mutated tumors		
				2 (20%) negative cases in 10 *TERT* mutated tumors		
Seok et al. [9]	24	H-score	RM236; monoclonal; RevMAb Bioscience; 1:10,000	Reduced in de novo and secondary ATCs	NA	No mutational analysis
	ATC					
	• 19 de novo ATC					
	• 15 secondary ATC					
The current study	318	H-score	Cat. no. 39791; polyclonal; Active motif; 1:10,000	Reduced in low-risk (NIFTP, FT-UMP, and WT-UMP) and malignant neoplasms (PTC, OCA, FTC, and IEFV-PTC) vs. benign neoplasms (FA and OA)	H-score	Cases with *TERT* promoter mutations showed lower 5hmC expression levels
	Thyroid neoplasms				Multifocality, bilaterality, invasive capsule status, major and minimal extrathyroidal extension, lymphatic and vascular invasion, biological behavior, and oncocytic morphology	Cases with *RAS* (*H*,* N*, or* K*) mutations, particularly *HRAS*, showed higher 5hmC expression levels
	• 14 OA			No 5hmC score difference between low-risk neoplasms (UMP and NIFTP) and minimally invasive, encapsulated angioinvasive, and widely invasive follicular pattern thyroid carcinomas		
	• 15 FA					No significant correlation was found between *BRAF* mutation and 5hmC expression levels
	• 3 WT-UMP					
	• 5 FT-UMP					
	• 18 NIFTP					
	• 14 IEFV-PTC					
	• 198 PTC and its subtypes					
	• 12 FTC					
	• 39 OCA					

Our results also showed a decreased level of 5hmC expression in the group of tumors with invasive/infiltrative growth patterns, with significant differences in intensity (*p* value < 0.001) and H-score (*p* value < 0.001). Interestingly, when we compared the invasive (*n* = 87) versus infiltrative (*n* = 102) growth patterns, 5hmC expression was similarly distributed across the score evaluations of intensity, extension, and H-score. A study by Seok et al. [10] focused on follicular-patterned thyroid neoplasms and grouped them as group I (NIFTP, 5 cases), group II (encapsulated FVPTC with capsular invasion, 14 cases), group III (infiltrative FVPTC, 10 cases), and group IV (PTC with a predominantly follicular pattern and well-formed papillae (< 1%), 11 cases). All cases were analyzed for 5hmC immunohistochemistry using the H-score, and 34 cases were evaluated for *BRAF* mutation analysis. 5hmC was highly preserved in groups I, II, and III, whereas group IV cases were noted with moderately reduced nuclear 5hmC [10] (Table 6).

In our cohort, *TERT* promoter mutations were found in 12 cases out of 183 PTCs, and 9 of them presented low H-scores (*p* value 0.003). Similar to our results, in 2020, Oishi et al. [11] showed that *TERT* promoter-mutated PTCs and ATCs have significantly decreased nuclear 5hmC levels in comparison with normal thyroid tissues and in PTCs with the wild-type *TERT* promoter. Later, the same authors added 63 PTCs with wild-type *TERT* promoter, ten PTCs with *TERT* promoter mutations, and four ATCs, to evaluate 5hmC expression by IHC, and no difference was found in the two groups [11]. A recent study of Hysek et al. [12] examined a dataset consisting of 26 FTCs, 2 FT-UMPs, and 1 OCA. However, they were unable to confirm previous observations in papillary thyroid carcinomas regarding the relationship between 5hmC loss and *TERT* mutations.

Concerning *RAS* family genes, in the same subgroup of our cohort, *RAS* and *HRAS* mutations were associated with the highest H-score, reaffirming the association of these mutations with better prognosis, as has been demonstrated in the literature [14] (Table 6).

The current analysis also verified for the first time an association between a decreased 5hmC H-score and both multifocality (*p* value 0.007) and bilaterality (*p* value 0.043). Once again, these results relate the loss of this marker with clinicopathological features associated with poorer prognosis and the need for more aggressive treatment [22, 23].

In our results, 5hmC expression was significantly higher when we compared benign tumors with low-risk (*p* value < 0.001) and malignant thyroid tumors (*p* value < 0.001), while no differences were observed when comparing low-risk tumors versus malignant tumors. Seok et al. [9] analyzed 5hmC location together with isocitrate dehydrogenase 1 (*IDH1*) mutations focusing only on an ATC cohort composed of 9 cases that occurred de novo and 15 ATCs that were derived from either PTCs (12 cases) or FTCs (3 cases). The H-score was markedly reduced in ATCs and moderately reduced in PTC and FTC components. In contrast, in the nonneoplastic thyroid, the expression was highly preserved. This study emphasizes the role of 5hmC in the multistep carcinogenesis of thyroid tumors, as has been shown in other human cancers. Additionally, the positioning of low-risk tumors closer to malignant tumors found in our study highlights the current concern with overdiagnosis and overtreatment in thyroid tumors. Since we could not demonstrate clear differences in 5hmC expression between low-risk tumors and minimally invasive follicular pattern carcinomas (IEFV-PTC, FTC, and OCA), it is not possible to use this marker to discriminate these two classes of neoplasms. The lack of differences with widely invasive follicular pattern-carcinoma cases can be attributed to the few cases with wide invasion (2 OCA cases) in our series.

None of the previously mentioned studies specifically addressed thyroid tumors with oncocytic morphology as a substantial dataset, as well as the accepted separate class of oncocytic tumors (OA and OCA). Our data revealed for the first time a link between 5hmC expression and oncocytic morphology.

In our cohort, 33% of the cases presented oncocytic morphology, including well-differentiated tumors with oncocytic morphology (*n* = 52) and oncocytic tumors (14 OA and 39 OCA), according to the 5th edition of the WHO Classification of Endocrine and Neuroendocrine Tumors. There was a strong statistical association between oncocytic morphology and lower 5hmC intensity, extension, and H-score evaluation (*p* values < 0.001, < 0.001, and 0.037, respectively). This association remained a trend in the Mann–Whitney test evaluation (*p* value 0.06), presenting an association between this morphology and lower H-score values. This leads us to hypothesize that perhaps the genetic variations/mutations in mtDNA and the metabolic alteration characteristics of these tumors may play a relevant role in this association [24–29]. Most probably, these disruptive mutations in mtDNA, characteristic of oncocytic tumors, can lead to significant changes in the levels of some oncometabolites such as α-KG and succinate, affecting the activity of cellular epigenetic regulatory enzymes. Interestingly, in ongoing work in our group, we found that in thyroid cancer cytoplasmic hybrid (cybrid) cell lines harboring mtDNA mutation significant changes in alpha-KG and succinate levels (data not shown). This has already been observed in pheochromocytomas and other tumor types, with mutations in *IDH*, *FH*, and *SDH*, in which DNA is globally hypermethylated, promoting a metastatic behavior [1, 9, 25, 27]. Also, global hypermethylation has also been described in well-differentiated thyroid neoplasm [30].

Thus, the low 5-hmC staining we found, particularly in oncocytic tumors, leads us to suggest that the low 5-hmC staining in these tumors may be reflecting the pronounced hypermethylation pattern of the tumors. However, further studies are needed to clarify the specific role that 5-hmC may play in thyroid tumorigenesis, particularly in the context of oncocytic tumors.

## Conclusion

The present study strengthens the association between the loss of 5hmC and adverse pathological and clinical characteristics of thyroid tumors as well as the value in segregating benign cases from low-risk and malignant cases. However, this marker does not allow us to distinguish between low risk and malignant or even identify cases with minimal and wide invasion. The specific epigenetic mechanisms behind 5hmC loss of expression need to be further investigated based on the phenotypic differences of thyroid tumors, particularly oncocytic tumors.

## Data Availability

Dataset was deposited in the files of “Cancer signaling and Metabolism” research group of i3s. Access to dataset used might be possible by contacting with corresponding author.

## Declarations

### Disclaimer

The funders had no role in the design of the study; in the collection, analyses, or interpretation of data; in the writing of the manuscript; or in the decision to publish the results.

## References

[1] Lian CG, Xu Y, Ceol C, Wu F, Larson A, Dresser K (2012). Loss of 5-hydroxymethylcytosine is an epigenetic hallmark of melanoma. Cell..

[2] Pronier E, Almire C, Mokrani H, Vasanthakumar A, Simon A, da Costa Reis Monte Mor B, (2011). Inhibition of TET2-mediated conversion of 5-methylcytosine to 5-hydroxymethylcytosine disturbs erythroid and granulomonocytic differentiation of human hematopoietic progenitors. Blood.

[3] Kraus TF, Globisch D, Wagner M, Eigenbrod S, Widmann D, Munzel M (2012). Low values of 5-hydroxymethylcytosine (5hmC), the “sixth base”, are associated with anaplasia in human brain tumors. Int J Cancer..

[4] Dong ZR, Zhang C, Cai JB, Zhang PF, Shi GM, Gao DM (2015). Role of 5-hydroxymethylcytosine level in diagnosis and prognosis prediction of intrahepatic cholangiocarcinoma. Tumour Biol..

[5] Tucker DW, Getchell CR, McCarthy ET, Ohman AW, Sasamoto N, Xu S (2018). Epigenetic Reprogramming Strategies to Reverse Global Loss of 5-Hydroxymethylcytosine, a Prognostic Factor for Poor Survival in High-grade Serous Ovarian Cancer. Clin Cancer Res..

[6] Liu J, Jiang J, Mo J, Liu D, Cao D, Wang H (2019). Global DNA 5-Hydroxymethylcytosine and 5-Formylcytosine Contents Are Decreased in the Early Stage of Hepatocellular Carcinoma. Hepatology..

[7] Zhang J, Han X, Gao C, Xing Y, Qi Z, Liu R (2018). 5-Hydroxymethylome in Circulating Cell-free DNA as A Potential Biomarker for Non-small-cell Lung Cancer. Genomics Proteomics Bioinformatics..

[8] Tong M, Gao S, Qi W, Shi C, Qiu M, Yang F (2019). 5-Hydroxymethylcytosine as a potential epigenetic biomarker in papillary thyroid carcinoma. Oncol Lett..

[9] Seok JY, Astvatsaturyan K, Peralta-Venturina M, Lai J, Fan X (2021). TROP-2, 5hmC, and IDH1 Expression in Anaplastic Thyroid Carcinoma. Int J Surg Pathol..

[10] Seok JY, Fan X (2022). TROP-2 and 5hmC expression in follicular-patterned thyroid neoplasm emphasizing tiny well-formed papillae. Ann Diagn Pathol..

[11] Oishi N, Vuong HG, Mochizuki K, Kondo T (2020). Loss of 5-Hydroxymethylcytosine is an Epigenetic Hallmark of Thyroid Carcinomas with TERT Promoter Mutations. Endocr Pathol..

[12] Hysek M, Hellgren SL, Condello V, Xu Y, Larsson C, Zedenius J, Juhlin CC (2023). 5hmC Immunohistochemistry: A Predictor of TERT Promoter Mutational Status in Follicular Thyroid Carcinoma?. J Histochem Cytochem.

[13] Baloch ZW, Asa SL, Barletta JA, Ghossein RA, Juhlin CC, Jung CK (2022). Overview of the 2022 WHO Classification of Thyroid Neoplasms. Endocr Pathol..

[14] Povoa AA, Teixeira E, Bella-Cueto MR, Batista R, Pestana A, Melo M (2021). Genetic Determinants for Prediction of Outcome of Patients with Papillary Thyroid Carcinoma. Cancers (Basel).

[15] Shi DQ, Ali I, Tang J, Yang WC (2017). New Insights into 5hmC DNA Modification: Generation. Distribution and Function. Front Genet..

[16] Kohli RM, Zhang Y (2013). TET enzymes, TDG and the dynamics of DNA demethylation. Nature..

[17] Hu L, Lu J, Cheng J, Rao Q, Li Z, Hou H (2015). Structural insight into substrate preference for TET-mediated oxidation. Nature..

[18] Xu T, Gao H (2020). Hydroxymethylation and tumors: can 5-hydroxymethylation be used as a marker for tumor diagnosis and treatment?. Hum Genomics..

[19] Barazeghi E, Gill AJ, Sidhu S, Norlen O, Dina R, Palazzo FF (2016). 5-Hydroxymethylcytosine discriminates between parathyroid adenoma and carcinoma. Clin Epigenetics..

[20] Zhang LY, Han CS, Li PL, Zhang XC (2016). 5-Hydroxymethylcytosine expression is associated with poor survival in cervical squamous cell carcinoma. Jpn J Clin Oncol..

[21] Tuttle M, Morris L, Haugen B, Shah J, Sosa J, Rohren E, et al. Thyroid-differentiated and anaplastic carcinoma (Chapter 73): Springer International Publishing; 2017.

[22] Lin JD, Chao TC, Hsueh C, Kuo SF (2009). High recurrent rate of multicentric papillary thyroid carcinoma. Ann Surg Oncol..

[23] Qu N, Zhang L, Wu WL, Ji QH, Lu ZW, Zhu YX, Lin DZ (2016). Bilaterality weighs more than unilateral multifocality in predicting prognosis in papillary thyroid cancer. Tumour Biol..

[24] Joshi K, Liu S, Breslin SJP, Zhang J (2022). Mechanisms that regulate the activities of TET proteins. Cell Mol Life Sci..

[25] Shi DD, Anand S, Abdullah KG, McBrayer SK (2023). DNA damage in IDH-mutant gliomas: mechanisms and clinical implications. J Neurooncol..

[26] Murugan AK, Bojdani E, Xing M (2010). Identification and functional characterization of isocitrate dehydrogenase 1 (IDH1) mutations in thyroid cancer. Biochem Biophys Res Commun..

[27] Xiao M, Yang H, Xu W, Ma S, Lin H, Zhu H (2012). Inhibition of alpha-KG-dependent histone and DNA demethylases by fumarate and succinate that are accumulated in mutations of FH and SDH tumor suppressors. Genes Dev..

[28] Maximo V, Lima J, Prazeres H, Soares P, Sobrinho-Simoes M (2012). The biology and the genetics of Hurthle cell tumors of the thyroid. Endocr Relat Cancer..

[29] Canberk S, Lima AR, Pinto M, Soares P, Maximo V (2021). Epigenomics in Hurthle Cell Neoplasms: Filling in the Gaps Towards Clinical Application. Front Endocrinol (Lausanne)..

[30] Keelawat S, Thorner PS, Shuangshoti S, Bychkov A, Kitkumthorn N, Rattanatanyong P (2015). Detection of global hypermethylation in well-differentiated thyroid neoplasms by immunohistochemical (5-methylcytidine) analysis. J Endocrinol Invest..

